# Supplementary material for: Content-rich biological network constructed by mining PubMed abstracts
Source: BMC Bioinformatics. 2004 Oct 8;5:147. doi: 10.1186/1471-2105-5-147 (PMC528731; doi:10.1186/1471-2105-5-147)
Supplement: Additional File 2 — The original results of the above study (non-essential files are deleted to keep the file size under the limit set by BMC bioinformatics). [file 1471-2105-5-147-S2.bz2 › chilibotAdditionalFile2/dip10/110ID8604139E454/html/FLIM_FLIN.html]

 


 **FLIM** and **FLIN** 
  
Found 43 abstracts in PubMed, retrieved 10.  
 

 What does Google say? 
 PDF only 
| .edu only 

---

**Interactive relationship** (e.g. stimulation, inhibition, etc)

**Non-interactive relationship** (e.g. studied together, co-existance, homology, etc.)

- Electron microscopic analysis indicated that polar and lateral flagellum specific FliG,  **FliM** , and  **FliN**  switch proteins are necessary for assembly of the respective flagella.  Ref: 11948156 J Bacteriol, 2002
- Both  **FliM**  and  **FliN**  possess charged domains not found in proton type homologs.  Ref: 10648530 J Bacteriol, 2000
- Incubation of basal bodies at pH 2.5 removed FliG,  **FliM** , and  **FliN**  but not FliF.  Ref: 11591685 J Bacteriol, 2001
- Incubation of purified basal bodies at pH 4.5 removed  **FliM**  and  **FliN**  but not FliG or FliF.  Ref: 11591685 J Bacteriol, 2001
- Phylogenetic analysis indicates that the polar and lateral FliG,  **FliM** , and  **FliN**  switch proteins are closely related and most likely arose as a gene duplication event.  Ref: 11948156 J Bacteriol, 2002
- Flagella purified from Salmonella enterica serovar Typhimurium contain FliG,  **FliM** , and  **FliN** , cytoplasmic proteins that are important in torque generation and switching, and FliF, a transmembrane structural protein.  Ref: 11591685 J Bacteriol, 2001
- To address this question, we purified the intact switch complex constituting the switch proteins FliG,  **FliM** ,  **FliN**  and the scaffolding protein FliF in quantities sufficient for biochemical work and used it to investigate whether or not the binding of CheY to the switch complex is cooperative.  Ref: 12736245 J Biol Chem< MedlineTA>J Biol Chem, 2001
- FliG,  **FliM** , and  **FliN** , key proteins for torque generation, are located in two rings.  Ref: 10468575 Proc Natl Acad Sci U S A, 1999
- The flagellar motor switch complex, consisting of the three proteins FliG,  **FliM** , and  **FliN** , plays a central role in bacterial motility and chemotaxis.  Ref: 10809678 J Bacteriol, 2000
- The basal body C rings, composed of the  **FliM** , FliG and  **FliN**  proteins, form the rotor of the flagellar motor 2.  Ref: 10959841 Curr Biol< MedlineTA>Curr Biol, 2000
- About 25 copies of FliG are present in a large rotor mounted assembly termed the switch complex, which also contains the proteins  **FliM**  and  **FliN** .  Ref: 12093724 EMBO J, 2002
- In this work, we identify the switch components, FliG,  **FliM** , and  **FliN** , of the sodium type motor.  Ref: 10648530 J Bacteriol, 2000
- Three Salmonella typhimurium flagellar motor proteins, FliG,  **FliM**  and  **FliN** , are required for the switching of rotation sense.  Ref: 10788321 J Mol Biol, 2000
- These basal bodies lacked C rings but had intact M rings, suggesting that  **FliM**  and  **FliN**  are part of the C ring but not a detectable part of the M ring.  Ref: 11591685 J Bacteriol, 2001
